# Annotations

**Published:** 1891-09-26

**Authors:** 


### Annotations.

Honesty and the Professions.

The *London Figaro* appears to be a little displeased with us because of the contention put forth in our Students' Number that medicine is an honest profession, and does not tempt men to depart from candour and simplicity so urgently as they are tempted in the church and in the law. Our contemporary appears to have misunderstood us. We did not affirm that doctors are honester than clergymen and lawyers; nor do we now set up any such contention. But we do affirm, and re-affirm, that in the art of medicine, when scientifically conducted, there is little temptation for the practitioner to be dishonest. The lawyer, by the very necessity of the case, must strive to win his client's cause, whether that cause be just or unjust. Similarly the clergyman of every faith and sect, holds a brief for his faith and sect, and is under every conceivable temptation to stand by and defend his own faith or sect at all costs. But what is the position of the doctor? He is called upon to bring back his patient's lost health, or to rescue his endangered life. This is his chief business; and it is impossible to conceive of any means which will secure this end, to which the least taint of immorality can attach itself. There are, it is true, occasions in the doctor's conduct of the external and financial part of his business, when dishonesty may serve his financial purpose better than honesty. But these are mere accidents, and of very rare occurrence. Our contention is that the essence of the doctor's daily work, which is that of restoring health and saving life, offers no temptation to dishonesty; whilst the essence of the lawyer's work, which is that of winning his client's cause whether right or wrong; and the essence of the clergyman's work, which is that of pleading the cause of his faith or sect, whether he can fully believe in it or not—does every day urge upon the lawyer and the clergyman very special temptations. That the lawyer and the clergyman, who are themselves honest, resist those temptations we are fully convinced, nor would we be so foolish and unjust as to make either the lawyer or the clergyman responsible for the peculiar circumstances which are inevitably inherent in his calling. What we do insist upon is, that medicine itself, apart from doctors, offers less urgent temptations to dishonesty than either of the other professions. Of course, as the result of this, the doctor cannot claim such high merit for his personal honesty as the lawyer or the clergyman can. On the other hand, the demerit of the lawyer or the clergyman who is submerged by the greater temptations of his calling is smaller than that of the doctor who fails to resist the lesser temptations of his. But, in truth, our discussion of the subject was purely academic and scientific, and in no sense personal. It was intended for the consideration of young men who had not yet decided which, if any one, of the three professions they would follow; and it seems to us that it is very desirable to bring forward such considerations at such a time.

One's Own.

A stout and prosperous-looking woman who appeared before one of the Metropolitan magistrates the other day on a charge of cruelty to her child, seemed to be amazed and indignant that she could not "do as she pleased with her own," as she expressed it. No doubt the woman was one of those foolish creatures who seek to atone for their want of intelligence by the tender of a double payment of impudence. But the question of property in children is one which physiology alone can settle. As a matter of fact, no parent can claim that a child is in any sense entirely his own. To begin with, when a man marries, especially if he be a working man, he has usually no desire for children; and they come into the world as the result of his marriage, in opposition, very often, to his most ardent wishes. Children that is to say, are a part of God's order, and could no more be born except in obedience to the everlasting laws which the Divine Mind has imposed upon the world, than the rain could fall upwards to the moon instead of to the earth. Moreover, the unkind parent, by his neglectful and cruel treatment of his offspring, after birth and during childhood, entirely fails to establish any property right in them. We believe it is profoundly true, and will be proved so in the final summing up of this world's affairs, that children are, in a special sense, the offspring of God. The woe denounced in the New Testament upon those who wilfully injure the tender and helpless bodies and minds of the young, has deep scientific warrant. Undoubtedly, it would be better for persons who are permitted to be parents, and who have not brains or heart enough to recognise the nature of the obligations thereby imposed upon them,—it would be better, we say, for them that a millstone should be tied round their necks, and that they should be cast into the midst of the sea. Nothing that has life in it can ever be called an absolute pcssession of any man's; still less anything that has life, and mind, and soul, as children have.

The last of the Demographers.

We have been favoured by the publishers with a copy of the volume which reports and winds up the proceedings of the last International Congress of Hygiene and Demography. Nothing is easier than to raise a smile at the gatherings, and sayings, and doings of enthusiasts. The man in search of an amusing paragraph requires no knowledge of the subjects discussed. Any indiscriminate gathering, or, for that matter, any carefully selected gathering, of thousands of human beings of both sexes, cannot but contain a large number of odd, not to say eccentric individuals, at whose expense it is easy to excite a hearty and harmless laugh. The members of the International Congress as a body were no exception to this law of things. Some of them were honest, capable, and justly distinguished persons in their own peculiar specialties; a few were men of world-wide reputation, whose names will live in history; many were harmless and self-pleased faddists; whilst the majority were unknown camp followers of the scientific army whose intentions, at any rate, were certainly good. The mere fact of the Congress and its meetings was a demonstration in favour of general wholesomeness, healthiness, and well-being. If the public mind grasped nothing else, it was compelled to master, at least, this amount of knowledge—that hundreds of the picked men of all civilised countries had discovered great and dangerous faults in our material civilisation, and were earnestly minded to mend them. The moral teacher has always aimed at laying a foundation in his pupils' mind of consciousness of wrong-doing. If the preacher can once get his hearers to see and admit that their ways have been foolish and wrong he sets to work with a cheerful heart to build upon that foundation a superstructure of reasonableness and virtue. The great Congress of Health, which has just met and parted, has undoubtedly convinced the world that it is often a foolish world in matters of health, and that it produces for itself a dangerous crop of sufferings, miseries, and injuries by its ignorance and indifference to the simple science of sanitation. The Congress discussed subjects of the widest and most far-reaching importance. A glance at the index of Dr. Wynter Blyth's book is almost appalling. Food Adulterations, the Rational Disposal of the Dead, Disease Districts, Meat Inspection, Open Spaces, Alchoholism, the Homes of the Working Classes, the Refuse of Large Towns, Tropical Climates for European Races, Drinking Water, the Diseases of Animals in relation to those of Man, Sanitation Afloat, Asiatic Cholera, Cancer, the Scientific Observation of Children, Hospital Organisation, Occupation, and Morality—these and a hundred other subjects of more or less daily importance to every class of the population, were discussed by men who are not only experts in knowledge and training, but enthusiastic advocates of their several kinds of knowledge. The Congress undoubtedly rendered a public service of the very highest importance to England and the world by its meetings and its general work. May we hint a single word which is not one of praise? The acting officials, of whom the chiefs at least were medical men, did not quite sustain the reputation of their country and their metropolis for courtesy and business efficiency. We could speak, indeed, somewhat strongly on these points from personal and other knowledge, but we are anxious to make excuses for inexperience in novel situations. We would, however, impress upon those comparatively young members of the medical profession who were in influential places, that inefficiency is never made more tolerable, to those who suffer from it, by discourtesy. A man should always be a gentleman, even if he sometimes be in a muddle. All that, however, is past, and we can cordially recommend Dr. Wynter Blyth's little volume as a not unworthy record of a great and important international and historical event.

